# A Critical Review of the Hydrometallurgy and Pyrometallurgical Recovery Processes of Platinum Group Metals from End-of-Life Fuel Cells

**DOI:** 10.3390/membranes15010013

**Published:** 2025-01-08

**Authors:** Sinikiwe A. Mvokwe, Opeoluwa O. Oyedeji, Mojeed A. Agoro, Edson L. Meyer, Nicholas Rono

**Affiliations:** 1 Fort Hare Institute of Technology, University of Fort Hare, Private Bag X1314, Alice 5700, Eastern Cape, South Africa; 2 Department of Chemistry, University of Fort Hare, Private Bag X1314, Alice 5700, Eastern Cape, South Africa

**Keywords:** platinum group metals, recovery techniques, proton exchange membrane, end-of-life fuel cells

## Abstract

Recently, the recovery of metals extracted from the spent membrane electrode assemblies (MEAs) of fuel cells has attracted significant scientific attention due to its detrimental environmental impacts. Two major approaches, i.e., pyrometallurgical and hydrometallurgical, have been explored to recover platinum group metals (PMGs) from used proton exchange membrane fuel cells (PEMFCs). However, the efficacy of these methods has been limited by the low concentrations of the metals and the high costs involved. Essentially, pyrometallurgical processes result in the evolution of harmful gases. Thus, the hydrometallurgical process is preferred as a suitable alternative. In this review, an overview of the application of pyrometallurgical and hydrometallurgical methods in the recovery of PGMs is presented. The health risks, benefits, and limitations of these processes are highlighted. Finally, the hurdles faced by, opportunities for, and future directions of these approaches are identified. It is envisaged that this review will shed light on the current status of processes for the recovery of spent PGMs and propel their advancement for effective recycling strategies.

## 1. Introduction

A fuel cell is an electrochemical device that generates heat and water using the chemistry of hydrogen and oxygen [1]. This device can be used to drive electrochemical processes and can effectively transform chemical energy into electrical energy without causing noise or air pollution [2]. There is significant interest in its commercialization, and in its replacement of the burning of fossil fuel for producing electric power in vehicles, thereby facilitating the lowering of greenhouse gas emissions and global warming [3]. Proton exchange membrane fuel cells (PEMFCs) are potentially the most potent electrochemical devices, among many kinds of fuel cell (solid oxide, molten carbonate, alkaline, phosphoric acid, etc.), due to their abundant energy supply, high efficiency, low emissions, and high power density [4,5]. They consist of membrane electrode assemblies (MEAs), the primary components of the fuel cells in PEMFCs, and they are crucial for enhancing the electrochemical processes of power generation by having a proton-conducting membrane inserted between the gas diffusion and catalyst layers [6]. However, MEAs are the most significant among the primary components, since they are made by integrating many materials, typically a polymer membrane, a catalyst layer, and carbon paper [7]. Significant components within MEAs include platinum nanoparticles embedded on a carbon substrate as the catalytic layer and a proton-conducting membrane, such as Nafion [8]. During the electrochemical processes inside the fuel cell, the platinum nanoparticles serve as catalysts, assisting in hydrogen oxidation at the anode and in oxygen reduction at the cathode [9]. On the other hand, the Nafion membrane functions as a solid electrolyte, allowing protons to pass while preventing electrons from flowing [10,11]. This setup facilitates effective proton mobility between the anode and cathode, generating electricity as a by-product [5]. Together, these components in the catalyst layer hold a crucial position in the basic function of the PEMFC by triggering chemical reactions and determining the overall performance of the PEMFC system [7].

**Figure 1:**
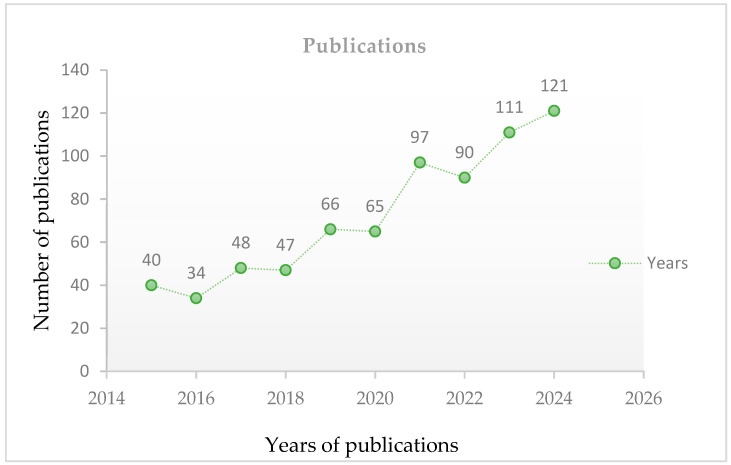
The number of articles published in 2015 covering the recovery of platinum. Data were obtained through Science Direct.

Platinum group metals (PGMs) are very scarce and unique metals in high demand in many industries. They are also used in fuel cells as catalysts. PGMs are naturally present in the Earth’s crust and are found in various locations, including South Africa. Anglo-American Platinum is the largest global producer, but it faces challenges in sustaining its position. Secondary material recycling and alternative processing methods, like the Kell process, can mitigate risks and ensure sustainable production [12,13,14,15,16,17,18,19,20]. According to reports, primary mining in South Africa boosted production from 163 tons in 2010 to over 200 tons in 2017, leading to increased energy consumption and pollution. As a result, research into secondary material recycling has long been seen as important, which has substantially stimulated its development as a steady supply of valuable materials [21]. As reported by Thomson Scientific’s Web of Science database, the number of publications released on recycling used catalysts has grown dramatically, from 18/year to 60/year. That is to say, the recovery of PGMs from trash is receiving intense and growing interest, and Figure 1 shows the number of publications on this topic from the past 10 years [22]. Existing research mostly focuses on PGM recycled from used catalytic converters, with minimal attention paid to catalyst reuse in fuel cell applications. However, recovery from automotive catalysts involves the use of harsh chemicals and energy-intensive methods, leading to potential environmental pollution and carbon emissions [23]. For example, Yakoumis et al. conducted a study on PGM recovery from spent automotive catalysts. In total, 90%, 92%, and 61% of the recovered Pt, Pd, and Rh were obtained via the leaching process. Because of the drawbacks of the pyrometallurgical process, Yakoumis et al. concluded that hydrometallurgical processes have the potential to become more widely used in industrial recycling through sustainable leaching design, which prioritizes gentler conditions, greener solvents, and high solid mass leaching [17]. In another study, Xu et al. presented the idea of recovering PGMs from spent catalysts following steps of crushing, roasting, reducing, and chlorination at 600–700 °C. However, this process is not suitable for industrial use because it emits toxic gases, requires a large amount of equipment, and operates at high temperatures [24]. Furthermore, Agnieszka et al. explored the recovery of PGMs from old automotive catalytic converters, utilizing a magnetohydrodynamic pump and lead as a metal collector. They identified challenges related to the efficiency of PGM elusion from the catalytic converters, noting that single flushing of the catalytic carrier with liquid metal only recovers a portion of the metals. Their studies employed 50,000 g of lead and examined how flushing durations changed platinum recovery rates from 40 s to 3600 s. Various secondary voltage settings (0.001 V, 0.16 V, and 0.26 V) were tested for their impact on platinum recovery efficiency, with 0.26 V resulting in higher recovery rates. The lead was held at 327.5 °C to ensure smooth metal flow and good contact with the catalyst surfaces. Lead (Pb) with purity level of 99.99% was selected as the metal collector because of its low melting point and effectiveness in removing PGMs from catalytic converters. When the concentration of PGMs in individual catalytic carriers is low, the process becomes economically unsustainable. The authors believe that using repeated flushes considerably increases recovery rates. The best strategy, according to their proposal, would be to submerge the catalytic converter carriers in this flow and flush continuously with liquid metal, which would increase the PGM recovery overall [25,26]. Therefore, it is of utmost importance to extract PGMs from end-of-life membranes, because they often hold a larger amount of PGMs compared to vehicle catalysts, making them a more promising reservoir of these crucial metals. Extracting PGMs from fuel cells can also lessen dependence on primary mining, enhance resource efficiency, and contribute to a more sustainable and circular economy. Moreover, extracting PGMs from fuel cells can aid in reducing the environmental impact linked with mining and processing these metals, further supporting the transition towards cleaner and greener technology. Hence, it is imperative to develop efficient and cost-effective methods for catalyst recovery, particularly hydrometallurgical technologies for reclaiming and reusing platinum from used MEAs, to alleviate the financial implications associated with platinum-based catalysts [27]. Herein, this review underscores the obstacles to, and possibilities and applications of pyrometallurgical and hydrometallurgical processes for PGM recovery from discarded proton exchange membrane (PEM) electrodes using non-corrosive, gentle chemicals. The health impacts and challenges are explored and identified. The prospects of these approaches are summarized. This review is anticipated to be of great benefit to scientists working on fuel cells and, thus, their commercialization.

### 1.1. Fuel Cells

**Figure 2:**
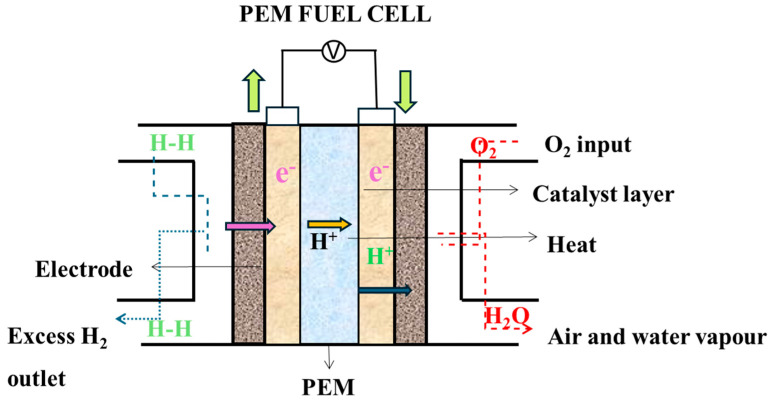
A typical assembly fuel cell.

The fuel cell is made up of the oxidant side (cathode), the fuel side (anode), the membrane that serves as a separator between the reactants, and an electrolyte to facilitate proton exchange. Figure 2 demonstrates the components of the PEM fuel cell.

### 1.2. Membrane Electrode Assembly

The MEA is an integral part of PEMFCs and electrolyzers, serving as a stacked structure comprised of various layers (shown in Figure 2) [28]. The MEA is a key component of PEMFCs [29], and it is typically composed of a Nafion ionomer, carbon support, and PGMs, mostly Pt, Ru, Ir, and PTFE (polytetrafluoroethylene), which offer electrolytes, carbon support, catalysts, and hydrophobic layer management, respectively. Depending on the formulation and manufacturing process, additional carbons and graphite may be present in tiny amounts [30]. Protons (H⁺) flow from the anode to the cathode via PEM, which prevents electrons (e^−^) from flowing in the other direction [31]. During fuel cell operation, the oxidation of hydrogen occurs at the platinum surface of the anode, resulting in the creation of H^+^ ions and e⁻. These electrons and protons then travel to the cathode via the membrane and the electrical circuit responsible for converting chemical energy into electrical energy. At the cathode side, oxygen is reduced and interacts with H^+^ ions to generate water. It is noteworthy that the reaction is exothermic, resulting in the generation of some heat alongside it [32]. Additionally, there are catalyst layers, which house catalyst particles, typically platinum contained within substances like carbon fiber paper to facilitate electrochemical reactions [32,33]. Another essential set of components is the gas diffusion layers (GDLs), which aid in delivering reactant gases (hydrogen and oxygen) to the electrodes [34].

#### 1.2.1. Proton-Exchange Membranes

The PEMs are semipermeable, reactant-barrier, and electronic insulators composed of ionomers that conduct protons [35]. They transport protons and separate reactants while blocking direct electrical channels in PEMFCs and electrolyzers [36,37]. PEMs can be constructed from composite membranes or pure polymer membranes. Nafion is a widely used material that is readily accessible on the market. Important properties of PEM include the proton conductivity (σ), thermal stability, and methanol permeability (P). The original PEM technology was developed in the 1960s by General Electric chemists Leonard Niedrach and Thomas Grubb, and NASA utilized it for the Project Gemini spacecraft program [38]. The popular medication Nafion was developed by DuPont polymers scientist Walther Grot [39]. Researchers investigated boron nitride and graphene atom-thick monolayers as potential alternatives for fluorinated ionomers in PEM materials When compared to other fuel cells, PEMFCs function at lower temperatures and are lighter and more compact, which makes them perfect for use in small devices and automobiles [40]. Recent developments in PEM fuel cell manufacturing techniques and essential materials show promise for sustainable energy applications.

#### 1.2.2. Catalyst Layer

In PEMFCs, the half-cell reactions occur in an active layer known as the catalyst layer, between the membrane and the gas diffusion layer. These layers contain catalyst particles (often platinum) embedded in materials like carbon cloth or carbon fiber paper. They facilitate electrochemical reactions [41]. The gas diffusion layer is covered with the catalyst layer or membrane to ensure close contact between the platinum or platinum alloy catalyst particles and the membrane. The design and elements of the catalyst layer are essential for maximizing the effectiveness and efficiency of the fuel cell [42]. The total electrochemical activity and longevity of the catalyst layer can be affected by variables such as catalyst loading, particle size, dispersion, and the porosity of the substrate [43]. Expensive platinum loadings were employed in the initial generation of PEM fuel cells. However, research has concentrated on lowering Pt loading to <0.4 mg/cm^2^ through improved platinum utilization techniques. Furthermore, effective catalyst recovery and re-use approaches can be very useful in further reducing the cost. Recent studies have shown that with platinum concentrations as low as 0.014 mg/cm^2^, catalyst cost is no longer a significant barrier to PEM fuel cell commercialization [44,45]. Optimizing catalyst layer properties, including ionic and electrical conductivity, hydrophobicity, and reactant diffusivity, is essential for maximizing the utilization of the catalyst material [46]. Moreover, a considerable obstacle to making PEMFCs commercially viable lies in the expense of cathode layers due to the use of expensive platinum metal as a catalyst. The ongoing quest for cathode layers with minimal or zero platinum content poses an intricate dilemma to overcome. Nonetheless, by modifying the properties of cathode layers, it is possible to enhance the catalytic efficacy while keeping platinum usage constant, thus leading to a reduction in the comparative cost of cathode layers [47].

#### 1.2.3. Gas Diffusion Layer

The GDL is a vital part of fuel cells. It serves as a permeable conductive material that improves the flow of reactant gases (hydrogen and oxygen) to the catalyst layer and the elimination of water produced during electrochemical reactions [48]. It plays a key role in ensuring efficient gas diffusion, providing the catalyst layer with mechanical support, and ensuring optimal fuel cell performance [49]. The GDL helps distribute reactant gases evenly across the catalyst surface, promoting optimal reaction rates. In addition, it facilitates the electron movement into and out of the catalyst layer [50,51]. The GDL regulates water by permitting an adequate amount of water to pass through and reside at the membrane for hydration. It is generally composed of porous carbon paper as the substrate or cloth, with the thickness ranging from 100 to 300 μm [52]. To prevent liquid water from filling the GDL pores, it is commonly coated with polytetrafluoroethylene (PTFE) Teflon [53]. This coating ensures that reactant gas transportation is efficient and the GDL functions effectively in maintaining the necessary balance of water within the system of fuel cells. Generally, the GDL is a vital part that improves the overall effectiveness and lifespan of PEMFCs [54].

Anode: *H*_2_ (*g*) → 2*H*^+^ (*aq*) + 2*e*^−^ (×2)  E^0^ = 0.00 V (1)

Cathode: *O*_2_ (*g*) + 4*H*^+^ (*aq*) + 4*e*
^−^ → 2*H*_2_*O* (*l*)  E^0^ = +1.23 V (2)

Overall Cell Reaction: 2*H*_2_ (*g*) + *O*_2_ (*g*) → 2*H*_2_*O* (*l*)  E^0^ = +1.23 V (3)

### 1.3. Classification of Fuel Cells

Generally, fuel cells can be categorized according to the type of electrolyte they use, as it is the main component that distinguishes different types of fuel cells, operational parameters, like temperature, or chemical parameters, like catalysts. The most common forms of fuel cells include alkaline fuel cells (AFC), proton exchange membrane fuel cells (PEMFC), phosphoric acid fuel cells (PAFC), solid oxide fuel cells (SOFC), and direct methanol fuel cells (DMFC) [55,56]. Table 1, below, shows a summary of fuel cells with various materials and operation temperature conditions.

## 2. The Physicochemical Properties of PGM-Based Catalysts in Fuel Cells

PGM-based catalysts are particularly successful in fuel cell electrochemical processes because of their superior stability, catalysis, unique adsorption properties, and electronic structure [59]. These catalysts ensure long-term performance under challenging working circumstances, such as high temperatures and acidic environments, by speeding up the rate of hydrogen oxidation and oxygen reduction [60,61]. PGMs also have unique adsorption characteristics that enable them to absorb molecules of reactants, such as oxygen and hydrogen, onto their surfaces and speed up the kinetics of reactions. The electronic structure of PGM-based catalysts is critical in defining their catalytic activity, since the density of states and distribution of electronic orbitals affect their interaction with reactants [61]. The energies involved in adsorption can be fine-tuned to increase the response speed by altering the surface of the atomic structure. The structure and morphology of the surface also affect electrochemical efficiency; catalysts with a high surface area and controlled morphology often exhibit improved activity due to increased exposure to active sites. As a result, techniques such as block copolymer lithography can produce nanostructured surfaces with increased electrochemically active surface area, as well as faster response times [62,63].

It can be mentioned that platinum is one of the heaviest metals and is known for its exceptional chemical and thermal stability [64]. These features have a crucial role in its widespread traditional uses and in the increasing number of new ones. Platinum has four unpaired electrons in its outermost shell, like other PGMs. However, these electrons reside in a d orbital, which is relatively far from the nucleus and weakly forms molecular bonds [64]. This unique electronic configuration allows platinum to participate in catalytic reactions without being consumed. Platinum can withstand high temperatures and harsh environments, ensuring its effectiveness over prolonged periods [65]. Its unique electronic structure, resistance to corrosion, and high-temperature stability contribute to its suitability as a catalyst in various industrial applications. This durability is crucial for applications like catalytic converters in automobile exhaust systems [66]. In catalytic converters, platinum makes the oxidation of hydrocarbons and carbon monoxide (CO) easier. Platinum excels under excessive-oxygen conditions, making it a preferred choice for diesel applications. It acts as a catalyst between the electrodes in fuel cells [67]. Hydrogen gas (H_2_) binds to platinum, breaking the H-H bond and generating two H⁺ ions. Its ability to bind hydrogen gas efficiently makes it an excellent catalyst for fuel cell reactions [68]. Other PGMs mentioned in the text also have interesting features that contribute to the overall efficiency, cost-effectiveness, and stability of fuel cells. For instance, ruthenium is known for its ability to improve efficiency by enhancing electrochemical reactions. Rhodium has excellent catalytic activity, and its corrosion resistance and oxidation enhance the durability of fuel cell components [69]. Iridium has excellent stability, it is the element in the periodic table that resists corrosion the best, and it can enhance the performance of fuel cells in harsh conditions. Palladium is less expensive than platinum, making it a more attractive option for large-scale use, and it is the most effective of the PGMs for reducing atmospheric pollution [70]. Rhodium is highly reflective and also exhibits excellent corrosion resistance [71]. Its catalytic properties are particularly valuable in automotive applications. Similarly to Pd, Rh is difficult to leach due to its noble nature and often requires aggressive leaching conditions. Ruthenium possesses distinct electrical attributes that render it useful for various catalytic applications, including fuel cells. Ru can form stable complexes with halides, making it more amenable to leaching compared to other PGMs, but still presents challenges due to its complex chemistry.

Pd is frequently used in place of Pt because of their similarities. However, Pt performs better than Pd in its pure state, and this equilibrium is upset when Pd is alloyed with transitional metals like Fe or Co. Pd is acclaimed for its outstanding catalytic performance and resistance to oxidation. It has a face-centered cubic crystal structure and exhibits high thermal and electrical conductivity [72]. The increase in catalytic activity is caused by a decrease in Gibbs free energy associated with electron steps, as well as a metal coupling effect, since Pd and transitional metals have low and high d-band occupancy, respectively. About 70–80% of Pd is utilized in the automobile sector as an active component of auto-exhaust catalysts. It is also used in terephthalic acid catalysts, vinyl acetate monomer catalysts, multilayer ceramic capacitors, printed circuit boards, and bonding wires [71]. The noble character of Pd makes it resistant to dissolution, necessitating strong acids or complexing agents for effective leaching. Iridium and osmium are utilized in different industries, primarily in the creation of crucibles suited to high-temperature environments, especially when alloyed with platinum to harden Pt, with an emphasis on fountain pen nibs [73]. These physicochemical properties contribute to iridium and osmium’s effectiveness in promoting efficient energy conversion in fuel cells.

Regarding the green chemistry principles, state-of-the-art leaching systems are based on mild solvents, lower temperatures, and direct processing. In several studies, a chlorine system seems favorable, as PGMs chloro-complexes are quite stable under acidic conditions.

## 3. Primary and Secondary Sources of PGMs

**Figure 3:**
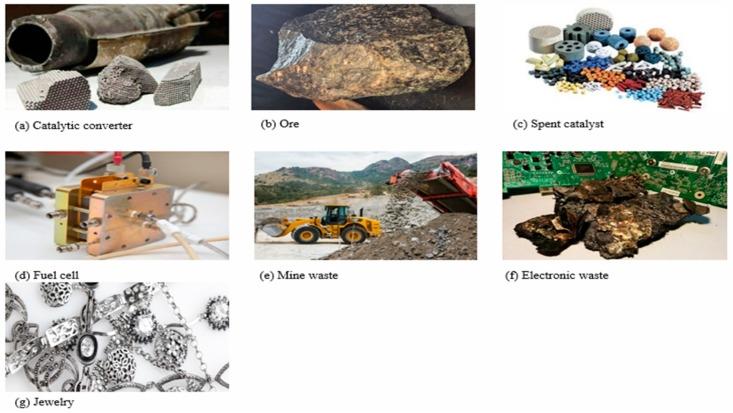
Platinum group metal sources (**a**–**g**) adapted from Google images. (**a**) https://www.privatefleet.com.au/blog/weird-stuff/boom-times-for-catalytic-converters/ (accessed on 1 October 2024), (**b**) https://www.finishing.com/439/83.shtml (accessed on 1 October 2024), (**c**) https://thepetrosolutions.com/feed-metals-hydrotreating-catalyst/ (accessed on 1 October 2024), (**d**) https://badgerchemistnews.chem.wisc.edu/2018/10/03/new-fuel-cell-concept-brings-biological-design-to-better-electricity-generation/ (accessed on 1 October 2024), (**e**) https://www.moneyweb.co.za/news-fast-news/worlds-top-platinum-miners-brace-for-substantial-wage-demands/ (accessed on 1 October 2024), (**f**) https://www.seprosystems.com/metal-recovery-from-weee-waste-electrical-and-electronic-equipment/ (accessed on 1 October 2024), (**g**) https://www.ecotradegroup.com/en/blog/we-market-sized-the-autocatalyst-recycling-industry-in-2017 (accessed on 1 October 2024).

PGMs are naturally found in small quantities in the Earth’s crust, primarily in sulfide and arsenide minerals [74]. Figure 3, below, demonstrates the sources of PGMs. They are recovered from secondary production through recycling processes or primary production, through mining activities [21,75]. Most PGM-containing ore reserves are found in ultramafic and mafic igneous rock formations in Canada, Russia, South Africa (Anglo-American Platinum), Zimbabwe (Great Dyke), and Ural–Alaskan-type complexes in Russia [73,76]. The Bushveld Complex in South Africa is the largest global source of PGMs, housing around 80% of PGM-bearing ore [77]. Its extensive reserves and well-established mining and processing infrastructure make it a dependable and significant source [78]. Anglo American Platinum is the foremost primary producer of PGMs worldwide, offering the advantages of scale, expertise, and a diverse range of products. However, it faces challenges in sustaining its position in a competitive and rapidly evolving industry and in addressing the ecological and economic impacts of its mining activities [15]. PGM mining is a complex, energy-intensive process with notable ecological impacts. However, when choosing a source, factors such as the specific PGMs needed, operational scale, geographical aspects, and environmental impact should be considered [19]. The Kell process, an alternative method for processing PGM concentrate, offers benefits like reduced energy consumption, cost savings, and lower CO_2_ emissions [18]. Adopting a diversified approach to sourcing can help to mitigate risks associated with reliance on a single source and confirm the long-term production of these precious metals.

Johnson Matthey’s 2024 PGM Market Report states that there will be a continuous demand for platinum, palladium, and rhodium that will outpace supply [79]. The platinum market is anticipated to see its greatest supply shortage in a decade. Despite a 2% expected drop in the primary supply, investments and industrial applications keep demand for platinum strong. The automotive industry, which is the most frequent user of platinum, has witnessed a slight decrease in usage, although it remains near its peak levels from the past decade [80]. The development of novel recycling processes is a key trend in PGM management to reduce expenses [81]. These techniques are dedicated to extracting PGMs from various waste sources, such as spent catalysts from fuel cells or automotive catalytic converters, more efficiently and cost-effectively. Moreover, ongoing research is being conducted on improving hydrometallurgical processes, solvent extraction techniques, and ion exchange methods to enhance the recovery efficiency of PGMs while minimizing operational expenses. The PGM industry is also witnessing a shift towards adopting circular economy principles, which involve closed-loop recycling systems and sustainable supply chain practices. This transition aims to improve cost efficiency and reduce dependence on primary sources of these precious metals [17].

**Figure 4:**
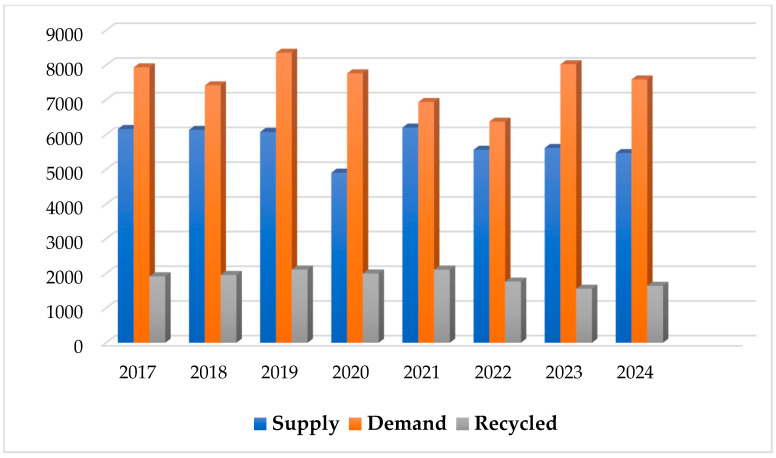
Annual comparison of platinum supply and demand developments from 2017 to 2024 with the recycled material (koz).

Sverdrup et al. used their models to study the full life cycle of PGMs and found that extraction would reach its maximum between 2020 and 2050, whereas market supply would reach its maximum between 2070 and 2080 [82]. Some studies utilized average catalyst recycling rates, whereas others relied on statistics from the US Department of Energy. None of these studies provide real-world data on the recovery of platinum and other PGMs after the end of their shelf lives. The supply of refined platinum varies yearly, as shown in Figure 4, below, with South Africa being the main growth driver. Despite seasonal constraints and a global recycling drop, the investment case remains attractive owing to resource shortages, strong demand, and a lack of supply. The International Monetary Fund (IMF) raised its expectations for global gross domestic product (GDP) growth to 3.2% in 2024, providing some upside to platinum demand [83,84]. Platinum mines are taking cost-cutting measures, delaying recovery. Investors are being informed on the investment case, with WPIC extending product partnerships and increasing interest in China.

## 4. Applications of PGMs on Catalytic Layers and MEAs

The characteristics of platinum (Pt) are strong supporters of its application in the catalyst layers of PEMFCs and are naturally used in various industries [85]. However, Pt tends to suffer from sintering which greatly increases its powder size, resulting in increased costs and seriously affecting its service life [86]. The increase in the utilization rate of Pt reduces its catalytic activity and ultimately affects the catalytic reaction of MEAs in PEMFC. Palladium (Pd) and ruthenium (Ru) are n-type agents. RuO_2_ has unique electrical, optical, and magnetic properties, as well as an extremely large dielectric constant [87]. It has been widely used in electrical materials and photoelectric devices and is considered an excellent ultrathin film electrolyte material due to its high electronic conductivity [88]. Electrodes, crucial parts of electrical devices that control the flow of electricity, are coated with platinum, palladium, rhodium, and iridium [89]. Additionally, flat-panel displays, cathode ray tubes, fiberglass, and liquid-crystal display (LCD) glass are made with platinum [90]. Ru is mainly used in the cathode gas diffusion layer or mixed with other platinum group metals to modulate thermal stability and reduce costs [91].

**Figure 5:**
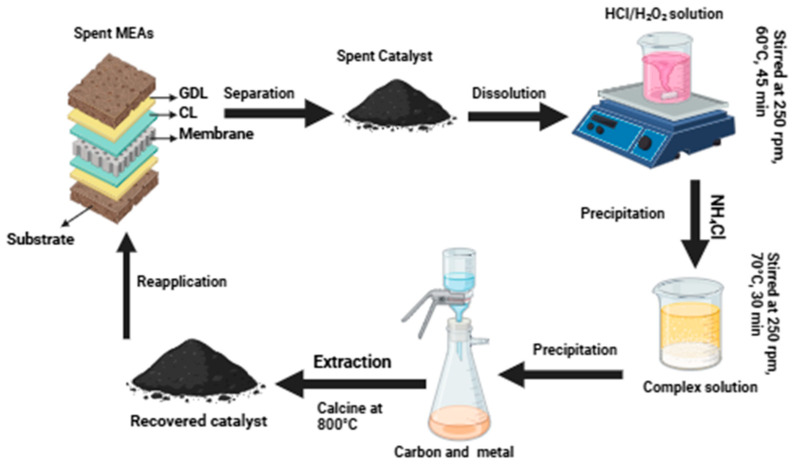
Recovery of PGMs and useability route for recycled material from used MEAs.

Pd is utilized in trace amounts to increase catalytic activity and has been shown to affect the resistance of catalyst materials to carbon corrosion in the catalyst layer [92]. In short, the modification of carbon support materials with Ru and Pd can help improve the dispersion, size distribution, and their interactions with Pt and, in turn, greatly improve the PGMs or Pt-based catalyst powder and the catalytic layer structure in the optimal catalytic system. The development of the latter is therefore beneficial for the widespread adoption of fuel cell technologies [93,94]. Figure 5 demonstrates a cyclic process of recycling PGMs from the spent MEAs of fuel cells. In the first step, the spent proton exchange membrane fuel cell stack is disassembled into multiple single cells and manually disassembled into bipolar plates and membrane electrode assemblies. This increases the surface areas of PGMs exposed to leaching agents, thereby improving recovery rates and resulting in more accessible metal ions for extraction during the leaching process, as revealed by other research [95,96]. Manual dismantling is time-consuming and inefficient, and limiting damage to the catalyst layer is critical for increasing recovery rates. Therefore, to delaminate the GDL of the MEA with the PEM, the MEA is cut into appropriate sizes, and 0.2 g of MEA is placed in 50 mL of a mixed alcoholic solution and deionized (DI) water (1:1, *v*/*v*) for sonication at 70 °C for 30 min. Next, a 100-mesh sieve is used to filter out the MEA fragments. Finally, the suspension is evaporated to dryness to obtain the spent Pt/C catalyst powder. For the dissolution step, a leaching solution of different concentrations is prepared in a 50 mL beaker and mixed with 1 g of the powder, and the resulting mixture is stirred while heated at 60 °C for 45 min to 1 h at 250 rpm. The samples are cooled and then filtered to remove the undissolved solids. In the precipitation step, a PGM-rich stream from the separation process is treated with NH_4_Cl to precipitate PGM in the form of (NH_4_)_2_PtCl_6_. Recovering a platinum salt seems to be more advantageous than recovering platinum metal, since the salt may be utilized to produce fresh catalyst. However, the stream can be filtered, washed with saturated NH_4_Cl solution, and dried to recover PGM in solid form. A step of ignition at 800 °C can be added to obtain high-purity PGMs (99.9%). Therefore, the recovered catalyst can be reused in new MEAs. The recovery of PGMs from MEAs is a rapidly evolving field, with improvements in technology and methodology. However, challenges such as inefficient dismantling, incomplete leaching, impurity separation issues, and high refining costs remain. Addressing these issues is critical to future sustainability and economic viability [97,98,99,100]. 

Platinum group metals, particularly platinum, play an important role in fuel cells, increasing efficiency and performance through their excellent catalytic characteristics, stability, and resistance to contamination [101]. Precious metals, especially Pt and Pt alloys, are crucial catalysts in the production of green electricity in PEMFCs by reducing oxygen and releasing water [101]. They are highly preferred as they are very active and stable over the long term, and they are perfect for optimum performance and dependability in PEMFC systems, as they are resistant to fuel cell impurities [102]. PGMs and catalytic layers find use in a multitude of sectors, including jewelry, transportation, portable electronics, and stationary power generation [71]. PGMs and catalytic-layered fuel cells are employed in stationary power production systems, such as installing backup power, telecommunications infrastructure, and remote locations [65]. They offer reliable and environmentally friendly energy solutions. They are employed in fuel cells for portable electronics, such as laptops, smartphones, and drones. These compact fuel cells provide a lightweight and long-lasting power source for on-the-go devices [103]. Fuel cells incorporating PGMs and catalytic layers are utilized in aerospace and military applications, including unmanned aerial vehicles (UAVs), satellites, and submarines. They offer high energy density and quiet operation, making them ideal for these specialized uses [104].

**Figure 6:**
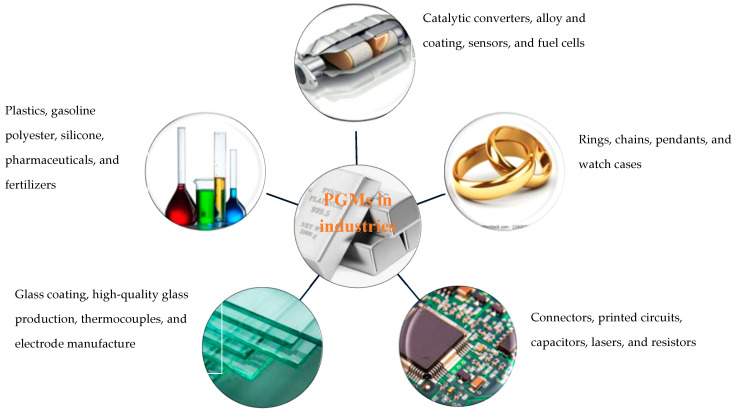
Uses of platinum group metals in various industries [85,110].

Platinum group metals (PGMs) are also crucial in industries like chemical, petroleum, automotive, jewelry, and medicine, as displayed in Figure 6. They are used as catalysts in refining crude oil, transforming it into essential products like gasoline, diesel, and jet fuel, and in environmental settings for gas cleanup, purifying harmful pollutants from industrial discharges [105]. Platinum is indeed a highly valued member of the platinum group due to its unique properties. In jewelry, luxury goods, and industrial uses, platinum is a highly prized substance due to its silvery-white appearance, scarceness, and resistance to tarnishing and corrosion [106]. Pt, Pd, and Rh can all work together to create ecologically friendly CO_2_, H_2_O, and N_2_ using automobile catalysts to transform the three dangerous gases CO, HC, and NO_X_ [107]. Pt and Pd achieve this by converting CO and HC into CO_2_ and H_2_O, and Rh by reducing NO_X_ to N_2_. Hence, automobile catalysts are occasionally referred to as three-way catalysts [23,108]. Particularly in acidic environments and high-temperature catalytic processes, PGMs are the most effective automatically distributed catalysts [109]. In conclusion, PGMs have a wide range of industrial uses, including in catalytic converters in vehicle emissions control systems, and as catalysts in chemical processes, electrodes in fuel cells, and components in electronic gadgets. The variety and efficiency of PGMs in various applications emphasize their relevance in current industrial processes, as well as their substantial contribution to technical innovation and environmental sustainability. Continued research and innovation in the use of PGMs are critical for improving their industrial uses and realizing their full potential advantages across several industries.

## 5. Metallurgical Extraction of PGMs from Fuel Cells

**Figure 7:**
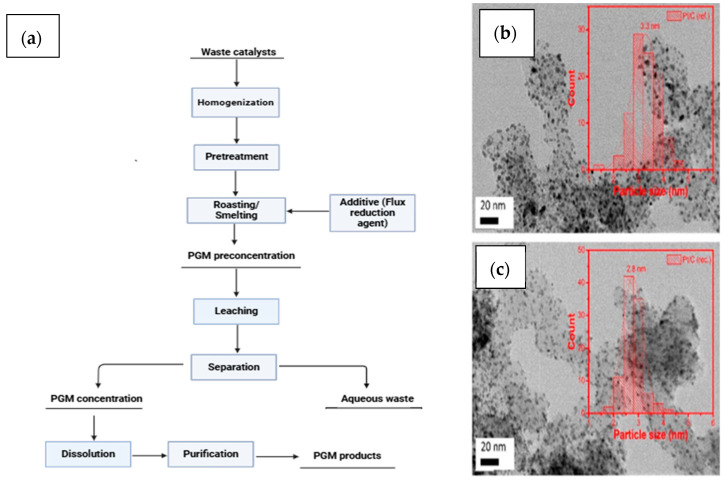
A flowchart diagram that shows pyrometallurgical steps for recovery of Pt catalyst from MEAs (**a**) with AFM images showing the size distribution of Pt nanoparticle baseline (**b**) and particles of recycled sample (**c**), adapted from Sharma et al. [134].

**Figure 8:**
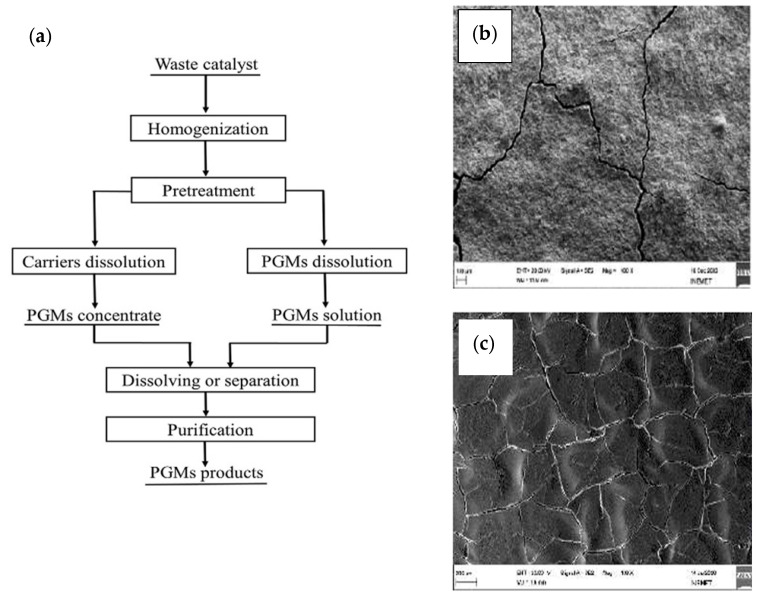
A flowchart diagram for hydrometallurgical process recovery of PGMs from waste catalyst (**a**) and SEM images of PtRu-based electrode material separated C-substrate from the electrode material (**b**,**c**), adapted from Sandig-Predzymirska, L., et al. [98].

Aged PEMFCs are collected and disassembled to isolate their components for recycling. The recovery of PGMs is promoted due to their high value and scarcity. However, the primary procedures for recycling PGMs from used catalysts using pyrometallurgical and hydrometallurgical methods are shown in the figures below (Figure 7 and Figure 8), and numerous other recovery strategies have been investigated. PGMs are extracted from membrane electrode assemblies using both physical and chemical techniques. The success of the recovery procedure is dependent on both the initial presence of precious metals in the MEAs and the efficacy of the extraction techniques used [29]. Depending on the particular refining techniques employed and the equipment in use, this process’ profitability may vary [111]. This article focuses on an advanced hydrometallurgical technology for the recovery of PGMs from spent PEMFCs. An acidic leaching solution with various oxidizing agents is used, and observations on temperature and time are noted. The reaction of PGMs with HCl and oxidizing agents results in the production of soluble chloro-complexes, and Equations (4)–(6), below, demonstrate the formation of complex PGM ions using HNO_3_ oxidizing agents [24]. These oxidizing agents cause HCl to decompose, generating Cl_2_ with PGMs while shortening the processing time by producing soluble PGM chloro-complexes (7 and 8).

Recently published papers on HCl/H_2_O_2_ leaching highlight the advantageous function of H_2_O_2_ as an oxidant, since it is more sustainable than HNO_3_ or other inorganic acids. The overall reaction of acidic leaching with H_2_O_2_ is presented in equation (**8**). This work focuses on using 37% hydrochloric acid, 50% hydrogen peroxide, and 10 vol% HNO_3_ to prepare leaching solutions. Furthermore, the HNO_3_ or H_2_O_2_ concentration is varied in the HCl-based leaching system to evaluate the influence of the oxidation agent on metal dissolution. The spent membrane from the Fuel Cell Store is soaked in an alcoholic solution for about 10 min to delaminate the catalyst layer and then dried to crush the catalyst powder out of the catalyst layer. The obtained powder is treated with an acid-leaching solution to extract platinum, while the remaining materials are left behind as solids in the residue. This can include polymers, such as fluoropolymers, or hydrocarbon types utilized in MEA fabrication. The platinum is then precipitated from the solution by adding NH_4_Cl, resulting in the formation of yellowish Pt precipitate (NH_4_)_2_[PtCl_6_]. The produced crude PGM salt is thoroughly removed from the reaction solution by washing it with a dilute NH_4_Cl solution (150 g/L). All of the necessary base metals and PGMs, Ir, Rh, and Pd, are to be separated are dissolved in the mother liquor, as well as Pt to some extent, and then subjected to calcination to obtain pure metals. Pure PGMs can be reused in many applications, such as counter electrodes for dye-sensitized solar cells to enhance their performance [112]. To guarantee excellent product purity, the contact period between the mother liquor and the precipitate must be kept short; otherwise, contaminants coprecipitate and contaminate the precipitate. Equation (9) shows the reaction that takes place.

3Pt + 18HCl + 4HNO_3_ → 3[PtCl_6_]^2−^ + 6H^+^ + 4NO + 8H_2_O (4)

Rh + 6HCl + HNO_3_ → [RhCl_6_]^3−^ + 3H^+^ + NO + 2H_2_O (5)

3Pd + 12HCl + 2HNO_3_ → 3[PdCl_4_]^2−^ + 6H^+^ + 2NO + 4H_2_O (6)

2HCl + H_2_O_2_ → Cl_2_ + 2H_2_O (7)

3HCl + HNO_3_ → NOCl + Cl_2_ + 2H_2_O (8)

Pt_(s)_ + 6HCl + 2H_2_O_2_ → [PtCl_6_]^2−^ + 2H^+^ + 4H_2_O (9)

3(NH_4_)_2_[PtCl_6_] → 3Pt + 16HCl + 2NH_4_Cl + 2N_2_ (10)

The selective extraction of these valuable metals is achieved while considering factors such as material composition, desired metal purity, and environmental concerns. This method aims to streamline the recycling process by eliminating the need for intermediate compounds, potentially enhancing efficiency and reducing operational complexities. Further exploration and advancement in this field could result in progress in the sustainable recovery of PGM and the synthesis of catalysts for PEMFC applications as demonstrated in Table 2.

### 5.1. Pyrometallurgical Process

Pyrometallurgical processing recovers precious and nonferrous metals from a variety of sources, including used automobile catalysts and electronic trash. It uses methods like roasting, smelting, sintering, melting, and high-temperature reactions [120,121]. The pyrometallurgical process is a common method used in industries to recover Pt due to its scalability and viability. Jia et al. [122] conducted a study focusing on extracting rhodium metal from used catalysts. They discovered that the Rh catalyst, which has a boiling point under 300 °C, makes up approximately 70 to 80% of the total material. When temperatures are kept below 300 °C, rapid heating can lead to the quick evaporation of the spent catalyst together with the release of organic substances. Before reaching 300 °C, the temperature was held steady for 1.5 h at 50 °C intervals. Subsequently, when it exceeded 300 °C, it was maintained for 1 hour at every increase of 100 °C, ultimately reaching a peak temperature of 1100 °C. Under these ideal conditions, the recovery rate of rhodium was found to be an impressive 99.9%. These methods are appropriate for devices with considerable fluorine content, such as PEMFCs, but not for devices with high fluorine concentrations due to the possible creation of hydrofluoric acid and other carbonyl fluoride (COF) compounds [123]. In addition, the high-purity content and 95% recovery rates present capability for extracting PGMs from these sources using a pyrometallurgical process. However, significant drawbacks, such as high energy consumption, possible environmental effects from emissions and waste products, low selectivity in extracting certain PGMs, and the possibility of losing precious metals during treatment, currently restrict the process [124]. Additionally, the pyrometallurgical recovery of PGMs from Nafion MEAs faces the disadvantage of emitting fluorihe at high temperatures. However, the application of inorganic additives has been proposed as a possible method for absorbing and regulating fluorine emissions during the high-temperature processing of Nafion MEAs. These organic molecules may chemically link with fluorine to form stable compounds that prevent HF from escaping into the atmosphere, thereby boosting the safety and sustainability of the pyrometallurgical process. Other methods include the adsorption of fluorine gas from aqueous solutions involving zeolites and van der Waals forces, with active sites for fluoride/fluorine uptake, the physorption of fluorine gas molecules, and investigating isotherms for fluorine and halogen adsorption. Combining these techniques may decrease environmental dangers and increase recovery process performance, assuring safety [125,126,127,128,129]. The recovery of Pd using pyrometallurgical processes results in a recovery rate of about 99%, while Rh can exceed 97% at high temperatures (1100–2000 °C). PGM alloy catalysts, such as PtPd and PtRu, increase catalytic activity and stability by utilizing distinct metal properties, possibly enhancing fuel cell performance via electronic interactions. According to the paper that Mohamad examined [108], thiosulfate leaching worked effectively for recovering Pd, with initial extraction rates ranging from 20% to almost 40% at concentrations ranging from 0.2 M to 0.6 M, but decreasing at higher concentrations owing to saturation. However, a controlled experimental setup utilizing a Plackett–Burman design revealed that under ideal conditions of (NH_4_)_2_SO_4_ 1.5 M, CuSO_4_ 0.03 M, Na_2_SO_3_ 0.1 M, Na_2_S_2_O_3_ 1.2 M, T (60 °C), and pH 8, up to 26.0% of Pd may be leached from synthetic catalysts (Pd/Al_2_O_3_) [108]. As a result, the presence of Pt might alter the leaching efficiency, thereby complicating the process of dissolution due to its reduced solubility. Rhodium and PGM alloy catalysts, such as PtPd and PtRu, frequently require aggressive leaching conditions because of their noble nature.

Horike et al. [130] introduced a chlorination method aimed at the efficient recovery of platinum (Pt). They employed CuCl_2_ as a chlorine source to treat Pt at temperatures between 400 °C and 600 °C. Because of its high chemical stability, pure platinum remained insoluble in hydrochloric acid (HCl) solution. To facilitate recovery, platinum-containing scrap was combined with magnesium, mixed with CuCl_2_ at 500 °C, and then dissolved in hydrochloric acid. Additionally, the high-temperature treatment of pyrometallurgy can cause the loss of volatile PGMs and other valuable components, which lowers recovery rates and metal purity. Due to the complexity of PEMFC materials and structures, pyrometallurgical processing may not be the ideal solution, since the high temperatures required may damage sensitive components and make PGM recovery difficult [17], and using pyrometallurgy is limited due to the high costs of equipment corrosion during evaporation [131,132,133]. Additionally, the emission of hazardous gases owing to energy consumption and fuel cell electrode compositions restrict the process. Thus, it is not a commonly preferred method for recovering PGMs from proton exchange membrane fuel cells (PEMFCs). Therefore, it is crucial to thoroughly explore these issues when considering pyrometallurgical processing for PGM recovery from MEAs. Hence, hydrometallurgical extraction is recommended for Pt recycling, with oxidant-assisted dissolution in chloride media being the most practical method due to Pt’s chemical features. This technology mitigates the environmental effect of harmful pollutants. Figure 7 represents the essential phases of pyrometallurgical procedures to recover PGMs from used catalysts together with results from previous studies.

### 5.2. Hydrometallurgy Process

Given the difficulties connected with pyrometallurgical recycling methods, hydrometallurgy has been considered as a recycling option. The hydrometallurgical process extracts metals from solid matrices by dissolving target components in aqua regia and acids, such as HNO_3_, H_2_SO_4_, HCl, or basic solution, in the presence of oxidizing agents, precipitating PGMs as intermediate products [135,136]. The major steps required for this procedure include leaching, solution concentration and purification, and metal recovery [29]. These steps are crucial in ensuring the efficient extraction and purification of metals from ores, concentrates, and recycled materials. This process effectively removes contaminants introduced through matrix dissolution, ensuring the purity of the recovered PGMs. Hydrometallurgy is particularly beneficial for recovering less reactive metals, like gold and silver, as well as for extracting metals from industrial by-products [137]. Hydrometallurgy is an efficient approach for extracting and purifying metals using aqueous solutions with low temperatures. It offers excellent metal selectivity, low energy consumption, and the possibility of reactant recycling [138,139]. However, it requires mechanical pre-treatment to increase the surface area, generate large solution volumes, and produce potentially toxic wastewater [140]. In contrast to the pyrometallurgical approach, hydrometallurgical processes are commonly used for PGM recovery from PEMFCs due to their ability to dissolve metals at low temperatures, allowing the selective extraction of PGMs and other valuable metals while minimizing the risk of damaging fuel cell components.

These processes are preferred for their efficiency and selectivity in recovering PGMs from complex materials like PEMFCs. The representation in Figure 8 shows the process of recovering the catalyst using hydrometallurgical methods, as well as outcomes from other articles. 

According to the available studies, leaching procedures for metal recovery frequently include the use of three primary inorganic acids: HCl, HNO_3_, and H_2_SO_4_ [141]. These strong acids effectively extract metals from materials, break them down, and release desirable metals. Alkaline conditions can result in the formation of metal oxides and hydroxides, which can inhibit leaching [142]. However, ammonia and other alkaline solutions can lead to reduced recovery rates because they are less effective in dissolving PGMs. Complexes such as tetraammineplatinum(II) [Pt(NH_3_)_4_]^2+^, triamminchlororhodium(III) [Rh(NH_3_)_3_Cl_3_], hexaammine–ruthenium(III) [Ru(NH_3_)_6_]^3+^, tetraammine–palladium (II) [Pd(NH_3_)_4_]^2+^, and diamminedichloroplatinum(II) [Pt(NH_3_)_2_Cl_2_] are examples of coordination compounds that can form with ammonia. However, their solubility and recovery efficiency may be compromised in alkaline conditions. Iron oxides can produce a passivating coating on the surface of the material, which prevents further leaching. Alkaline leaching techniques can be complicated and costly due to additional waste processing and neutralization operations. Although they are less corrosive and harmful than strong acids, they can harm the environment if not properly controlled. Proper disposal of alkaline waste is critical to avoiding contamination [143]. The referenced investigations demonstrated that the Pt anion has a very strong affinity for ammonium/amine. After the separation, platinum can eventually be recovered in solid forms, such as ammonium hexachloroplatinate (NH_4_)_2_PtCl_6_, by precipitation, utilizing ammonium chloride as a precipitation reagent [122].

Aberasturi et al. [144] stated, in their study, that 95% of PGMs could be recovered from used catalysts by dissolving them in an HCl, H_2_SO_4_, and H_2_O_2_ leaching solution at 90 °C for 6 h. They performed a thermal pre-treatment at 250 °C for 22 h to enhance the extraction efficiency and optimize the process. Their study discovered that under optimal circumstances, a considerable percentage of PGMs such as platinum, palladium, and rhodium may be recovered from used catalysts, with less aggressive reagents having a lower environmental effect. Yakoumis et al. examined a hydrometallurgical process using HCl, NaCl, and H_2_O_2_ and discovered that Pt, Pd, and Rh had recovery rates of 100%, 92%, and 60%, respectively [145]. Nevertheless, Paola Trucillo and his team [73,145] introduced a hydrometallurgical process for extracting platinum from diesel catalysts. In the first step, they immersed the catalyst in a mixture containing 0.2 M of H_2_O and 0.4 M of HCl. They observed that lower HCl concentrations resulted in extended leaching times. However, these conditions can be adjusted to obtain a successful recovery of platinum, while minimizing the extraction of other metals. The ideal conditions for the leaching process were found to be a temperature of 20 °C using 0.13 M H_2_O_2_ and 0.4 M HCl solution [81,145]. Paiva and colleagues extracted 84% of Pd using a leaching solution of HCl (2 M) and H_2_O_2_ (1 M) at 25 °C for 1 h. After 10 min of reaction, the oxidizing agent H_2_O_2_ sped up the dissolution. A similar leaching system was studied, yielding notable recovery rates of Pt 88%, Pd 99%, and Rh 77%. These rates appear to be enhanced by partially substituting NaClO (3%vol) for HCl (5 M), resulting in lower-acidity conditions without compromising the dissolving efficiency [146]. There is minimal research dedicated to Pt recycling from wasted PEM fuel cells. Zhao et al. [147] recovered Pt from PEMFC Pt/C catalysts by drying them at 80 °C for 3 h, and then igniting them at 600 °C for 6 h. They used aqua fortis to leach the residue from the catalyst, and then added HCl and heated the solution at 110 °C to convert nitrate ions into NOx gases. H_2_PtCl_6_ was recovered through evaporation, purification, and filtration. Pt/C catalysts were produced afresh from the recycled Pt using two suggested procedures. In another study [131], Xu et al. employed sulfuric acid as an oxidizing agent to separate fuel cell assemblies. They discovered that the key to recycling fuel cell assemblies is the effective separation of the membrane and catalyst layers, followed by the precious metal and acid resin layers. The process involves soaking a catalyst coated membrane in 0.5 M H_2_SO_4_ for 2 h, drying it in an oven at 80 °C under vacuum, and crushing the fragments into 0.5 cm^2^ samples for testing. After 72 h of treatment with concentrated sulfuric acid at 150 °C and under continual stirring, a clear solution with a black sediment at the bottom of the reaction vessel was formed. Atomic absorption spectroscopy revealed 7.33 mg/L of platinum, indicating that it was the quantity oxidized. This process produced 231.7 mg of recovered platinum, representing a 94.2% recovery rate, confirming its efficacy and simplicity. The work proved the effective treatment of acid processing for MEAs [122,148]. In the study conducted by Abha Bharti and his colleagues, the recovery of Pt/C catalysts and a Nafion membrane from end-of-life MEAs using a low-temperature hydrothermal treatment and a 50:50 *v*/*v* mixture of water and isopropanol (IPA) under ambient pressure was discussed. In that study, the aqueous IPA solution facilitated the disruption of bonds between the fluorocarbon-containing Nafion membrane and the coated Pt/C catalysts, allowing for catalyst detachment. The Nafion was subsequently dissolved, enabling recovery via vacuum filtration. As a result, a total of 0.74 g, representing a recovery rate of 98.7%, was recovered from the untreated EoL MEAs. The Pt content in the recovery catalyst was approximately 17 wt%. The ionomer solution was heat-treated at 80 °C for 12 h, producing ionomer powder that may be reused and dissolved in a variety of solvents [114].

An aqua regia, which is the combination of concentrated HCl and HNO_3_ at a ratio of 3:1, is commonly used for PGM oxidation and dissolution due to its ability to provide a high concentration of Cl^−^. In 2020, Yuliusman et al. carried out a study on the platinum recovery process using oxalic acid and aqua regia leaching on a used continuous catalyst regeneration (CCR) platform [149]. The experiment utilizing oxalic acid for leaching exhibited a maximum efficiency of 5.58%, but the trial utilizing aqua regia showed a 19.72% efficiency. The authors noted that even though organic acid is better for the environment, it is not as strong or efficient as aqua regia. The number of acidic anions generated increased with the number of organic acids, leading to a reduced leaching efficiency of 5.58%. Rzelewska et al. studied the recovery of PGMs, particularly platinum and rhodium, from used automobile converters using hydrometallurgical procedures such as leaching and liquid–liquid extraction [150]. Their work concentrated on using aqua regia with a solution of HCl, H_2_SO_4_, and H_2_O_2_ to leach Pt(IV) and Rh(III). The study revealed that the Pt(IV) and Rh(III) concentrations increased with time, with both the aqua regia and the HCl/H_2_SO_4_/H_2_O_2_ mixture having leaching efficiencies of no more than 35%. It was discovered that using HCl/H_2_SO_4_/H_2_O_2_ instead of aqua regia worked well, creating no harmful gases like NOx while maintaining a comparable leaching efficiency [151]. Strong oxidative conditions are crucial for the dissolution of PGM in automotive catalysts, as the study emphasizes. Metal recovery from ores and waste materials is critical to industrial efficiency and economic viability. As raw material costs rise and demand for environmentally friendly practices grows, economic inquiries into metal recovery processes are critical. Effective decision-making for stakeholders is facilitated by striking a balance between cost-effectiveness, efficiency, and environmental impact. By concentrating on cost-effectiveness, efficiency, and comparative assessments, stakeholders may improve profitability and sustainability in metallurgical operations. Table 3 shows the full cost for the 500 kg batch of metal that was recovered. The metal recovery from the 500 kg batch of wasted catalysts showed good cost feasibility, according to the economic evaluation. Each batch of the process generates a net profit of USD 1158, with USD 2278 in total revenues (from recovering 81.6 kg Mo and 68.0 kg Co) offset by USD 1120 in total operating costs, for a profit margin of around 51% on. Despite extensive investment in chemicals, equipment, and labor, the high market value of recovered metals ensures successful operations, with a 103% return on investment for every batch [152].

The use of aqua regia poses environmental concerns as it can lead to the formation of harmful by-products, such as NO, NOCl, Cl_2_, and HCl(g) [153]. While aqua regia is effective in dissolving gold, it has a lower extraction rate for Rh compared to halogen oxidants like Cl_2_ [154]. Both Cl_2_ and HNO_3_ are corrosive reagents with handling and environmental issues. To address these challenges, alternative approaches, such as multi-stage leaching, pre-oxidation, and pre-reduction, are being explored [155]. The use of H_2_O_2_ as an oxidant in leaching metals may not offer significant benefits compared to O_2_ (g), while the potential of O_3_ as an oxidant remains under investigation [156]. It is also noted that HCl is not the only lixiviant used in PGM leaching processes, with other halogens and cyanide being utilized in some cases.

The use of hydrometallurgical processes is preferred due to their focus on sustainability, efficiency, and flexibility in handling PGMs. In the study proposed by Duclos et al., [154] hydrometallurgy has been recommended to remove Pt from the catalyst-coated membrane (CCM) MEA of a PEM fuel cell system. The authors managed to recover 76% of the Pt, and more processing was required to handle the solid waste and minimize the HCl. Existing approaches for recycling valuable electrocatalysts (Pt, Ir) used in PEMFC systems are often inefficient due to design restrictions, inadequate recycling technology, and separation thermodynamics. Therefore, research development is required to increase the recycling efficiency and efficiency of these valuable electrocatalysts. Most PGMs are extracted from discarded catalytic converters using a variety of metallurgical and refining procedures, such as smelting furnaces, hydrogen treatment, and direct leaching of platinum group metals. However, one significant problem is the inert nature of PGMs, which renders them resistant to ordinary acids [96]. To solve this, significant amounts of a powerful oxidizing agent (such as nitric acid, chlorate, hypochlorite, perchloric acid, bromate, nitrate, or cupric ions) combined with a strong acid (like HCl) must be used over a prolonged period to dissolve the PGMs. Therefore, the purpose of this paper is to provide a thorough evaluation of the existing level of understanding regarding the hydrometallurgical processing of spent catalysts through acid leaching and their ultimate recovery through solvent extraction. This will serve as a foundation for evaluating the efficacy, efficiency, and environmental implications of these processes while recovering PGMs.

## 6. Global Status of PGMs

**Figure 9:**
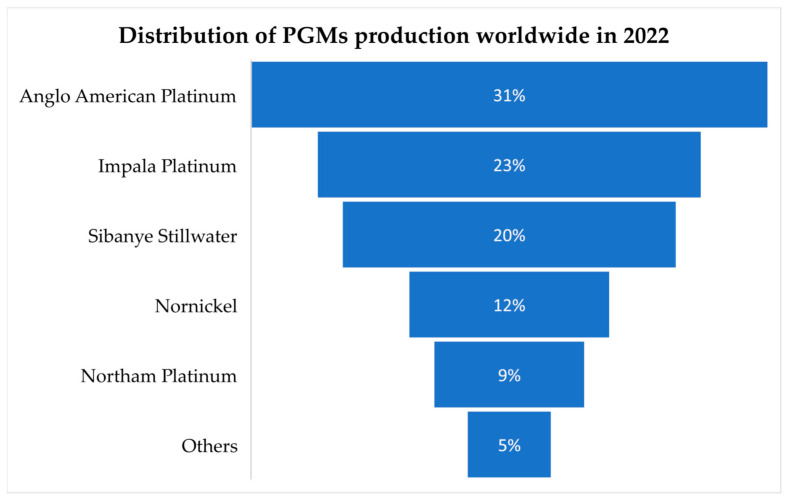
Top five platinum group metal manufacturing companies worldwide in 2022.

**Figure 10:**
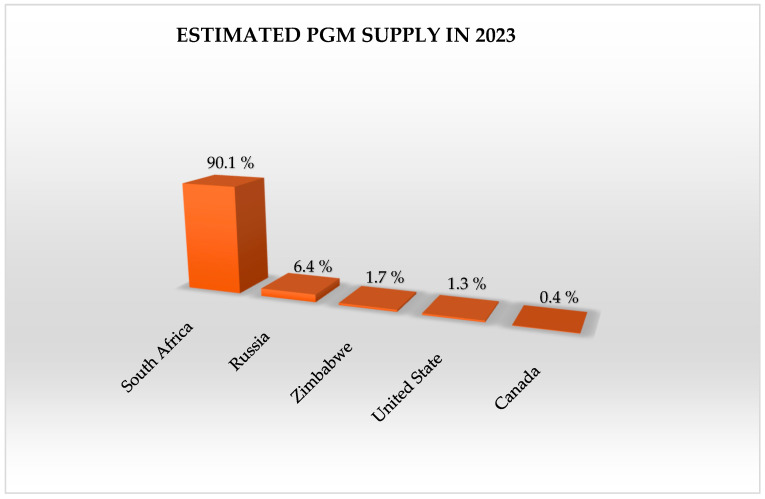
Estimated PGM supply for top 5 countries in 2023.

South Africa currently holds the top position as the leading producer of platinum group metals (PGMs), with 76% of the world’s platinum, 33% of its palladium, and 82% of its rhodium production [157]. This dominance is evident through its production of 140 metric tons in 2022 and 120 metric tons in 2023 [158]. However, challenges such as adverse weather conditions, safety shutdowns, labor disputes, and skill shortages have led to decreased output from major mining companies like Sibanye-Stillwater, Impala, and Lonmin [159]. Consequently, the global supply chain for a significant portion of rhodium and platinum is at risk, as one nation holds a near-monopoly on these precious metals. However, Anglo-American Platinum was one of the industry’s leading participants in South Africa, producing approximately 50.46 metric tons of platinum in 2022, as illustrated in Figure 9. On the other hand, Russia emerges as the second-largest producer, although with a lower output, of approximately 20 metric tons in 2022 and 23 metric tons in 2023 [160]. The company, previously recognized as Norilsk Nickel, achieved a significant milestone in 2022 by producing 16.81 metric tons of platinum, solidifying its position as a leading global producer of the metal. In contrast, platinum mine production in the United States has seen a downward trend in recent years, dropping to approximately 2.9 metric tons in 2023 [161]. Figure 10 shows that the top five platinum-producing countries in 2023 were South Africa, Russia, Zimbabwe, Canada, and the US, collectively contributing to 96% of the global platinum production share [16]. By 2030, worldwide platinum production is expected to increase by 1.3% per year, reaching 187.12 metric tons [162]. South Africa is likely to remain the leading platinum provider, producing 127.57 metric tons by 2030. Other notable producers include Canada, Zimbabwe, Russia, and the United States. Nevertheless, because of decreased output from South Africa and Russia, worldwide production is predicted to fall by 1.5% in 2024, while production in the US is predicted to increase [73]. However, output is predicted to rebound, with a 3.6% growth rate in 2025, reaching 178.60 metric tons, led by further growth at South Africa’s Two Rivers mine and the start of the Mareesburg project [161].

## 7. Environmental and Health Impacts of PGMs

Platinum recycling is a promising approach for reducing PEMFCs’ environmental effects, but its appropriateness has not often been considered due to a lack of accurate information. Pehnt [162] proposes a 75% recycling rate. However, it is unclear whether the accompanying implications were examined in his sensitivity studies. As shown in Figure 5, there has been a high demand for and short supply of PGMs since 2017. Studies on sustainable PGM consumption have been carried out, with the analysis by Saurat and Bringezu in 2008 [163] reporting that primary production in Europe has far more drastic ecological impacts than secondary production, with increased sulfur dioxide and carbon footprints [164]. A recycling technique for the platinum catalyst used in fuel cells was evaluated in the environmental study by Duclos et al., which discovered that recovering platinum can prevent more than half of the major effects of the membrane electrode assembly life cycle [164]. The extraction and process of mining PGMs lead to the destruction of habitats and water pollution. Hence, they require a large amount of land and water resources [165]. However, the refining processes and burning of PGM-containing products, such as catalytic converters in vehicles, release dangerous substances, emitting toxic chemicals into the environment, contributing to air pollution and climate change, putting wildlife and nearby communities at risk [166]. Moreover, the detrimental effects on health from exposure to PGMs are substantial, particularly impacting residents and individuals working in the mining, refining, and manufacturing industries [167]. Workers in these sectors may experience respiratory complications, skin irritation, and other health issues due to occupational exposure to these metals [168,169,170]. Previous research compared the environmental effect of PEM fuel cell life cycles to that of internal combustion engine automobiles. The energy-intensive procedures involved in PGM production also contribute substantially to environmental impacts, with mining and beneficiation playing a major role in the overall environmental burden.

## 8. Conclusions and Prospects

This review examined novel techniques used to recover PGMs from end-of-life PEMFCs while evaluating the recovery processes. The recovery of PGMs involves several hurdles, including low costs, environmental issues, and separation complexity. These challenges can be solved by investigating potential solutions, such as creating more effective extraction technologies, utilizing environmentally friendly processes, such as hydrometallurgy, and adopting sophisticated separation procedures, like solvent extraction or ion exchange. The development of procedures and the establishment of supporting infrastructure for recycling operations have led to significant economic and academic advancements. Academic research has concentrated on developing acid leaching for PGMs, while pyrometallurgical–hydrometallurgical recycling dominates the industry’s operating environment. Collaboration among academics, business, and government organizations is critical for creative thinking and defined standards, which promote sustainable practices in the fuel cell sector. The cost of hydrometallurgical processes is directly impacted by the number of recovery stages required, which is correlated with the amount of waste generated. The conditions used by hydrometallurgical processes are crucial for PGMs and result in excellent recovery. Mild leaching systems reduce liquid waste while also benefiting the environment and working conditions by not emitting toxic gases or byproducts. This review highlighted PGM recovery techniques with favorable conditions and better yields. More research ought to integrate energy and economic data to conduct a thorough techno-economic evaluation, enhancing the instrument for creating sustainable recycling techniques.

## Figures and Tables

**Table 1:** Classification of fuel cells [57,58].

Properties	PEMFC	PAFC	AFC	DMFC	SOFC
Operating temperature	50–80 °C	180–210 °C	65–220 °C	50–120 °C	800–1000 °C
Electrolyte	Solid polymer membrane	Phosphoric acid (H_3_PO_4_)	Potassium hydroxide aqueous	Polymer	Ion-conduction ceramic
Catalyst	Platinum or carbon	Carbon	Platinum or nickel	Platinum and others	Perovskites
Efficiency	45–55%	40–45%	60–70%	25–40%	>60%
Application	Automobiles	Stationary power stations	Space shuttle	Battery replacements	Large utilities
Power range	1–100 kW	<200 kW	1–100 kW	0.5–1 kW	<100 kW
Benefits	Environmentally friendly, low temperatures	Tolerant of CO_2_	High efficiency	Low temperatures, high power density	Non-precious metal catalyst, no CO or CO_2_ emission
Limitations	Sensitive to CO, uses Pt catalyst	Low efficiency, greenhouse gas emission	Sensitive to CO_2_	Sensitive to impurities, low efficiency	High temperatures, expensive ceramic material

**Table 2:** A comparative table for recovery rates of PGMs from MEAs of fuel cells using various techniques.

Approaches	Catalyst Source	Pathways	Recovery Efficiency and Metals	References
Hydro-leaching processes	Spent Petroleum Catalysts	Aqua regia	Pt 97%	[113]
High-temperature combustion	PEMFCs	Hydrothermal treatment	Pt, 98.7%	[114]
Hydrometallurgical method	PEMFCs	Acid dissolution	94.2%	[115]
Electrochemical-assisted dissolution	PEMFCs	Halide solutions (Iodide)	>99% for Pt, 37% for Ru	[116]
Oxidative nitric acid pre-leach	UG-2 concentrates	HNO₃ + HCl/Cl₂	>90% for Pt, Pd	[117]
Platsol process	UG-2 concentrates	H₂SO₄ + NaCl	Up to 98% for Pt, 95% for Pd	[117]
Hydrometallurgical method	Spent Auto-Catalysts	Hydrochloric acid leaching	90–98% Pt, 99% Pd, 70–96% Rh	[81]
Hydrometallurgical method	PEMFCs	Hydrochloric acid leaching	Pt 90%	[118]
Electrochemical dissolution	MEAs of PEFCs	H₂SO₄ + HCl	93.2% for Pt, 98.4% for Ru	[119]

**Table 3:** An economic analysis of metal recovery from wasted catalysts.

Item	Price in USD	Quantity
Spent catalyst	120	500 kg
Hydrogen gas	34	65 cm^3^
Energy	63	2114 kWh
Nitric acid	30	22 cm^3^
Power	58	1647 kW
Sulfuric acid	22	70 cm^3^
Ammonia	84	300 cm^3^
Hydrochloric acid	6	5 cm^3^
Nitrogen gas	12	80 cm^3^
Subtotal	448	
Consumption rate	64	
Accessories	90	
Equipment	112	
Maintenance	35	
Spare parts	85	
Service	35	
Other	24	
Subtotal	445	
Overhead charges	7	
Labor	116	2 weeks
Banking rate	10	
Losses	32	
Other	62	
Subtotal	227	
Total	1120	
Profit		
Co metal	966	68 kg
Mo metal	1312	82 kg
Total profit	2278	
Net profit	1158	
